# Serum Creatine Phosphokinase Level as a Prognostic Marker for Organophosphorus Poisoning and Its Correlation With Peradeniya Organophosphorus Poisoning Scale

**DOI:** 10.7759/cureus.88332

**Published:** 2025-07-19

**Authors:** Venkatesh Yellapu, Debasis Pathi, B Rajendra Prasad Rao, Lalatendu Mohanty, Sarada P Suna, Miriyala S Srinivas, Dipleshdeep Goyal, Shivam

**Affiliations:** 1 Cardiology, Government Medical College, Jammu, IND; 2 General Medicine, Kalinga Institute of Medical Sciences, Bhubaneswar, IND

**Keywords:** acute op poisoning, creatine phosphokinase (cpk), outcome, peradeniya organophosphorus poisoning (pop) scale, prognosis, severity of disease

## Abstract

Introduction: Organophosphorus (OP) compounds are widely used as insecticides in agriculture. The Peradeniya Organophosphorus Poisoning (POP) scale is a simple and effective system for determining the severity of OP poisoning. An increase in serum creatinine phosphokinase (CPK) levels in OP poisoning can be due to persistent oxidative cellular damage to muscle membranes. Accordingly, this study was conducted to assess the correlation between serum CPK levels and the severity of acute OP poisoning.

Aim: This study aimed to correlate serum CPK and disease severity with respect to the POP scoring scale, pseudocholinesterase levels, the requirement for atropine, and the clinical outcomes of the disease.

Materials and methods: This observational study involved 60 patients with OP poisoning admitted to a tertiary care setup from September 2018 to August 2020. The patients were clinically assessed and categorized based on the POP scale at the time of admission. Serum CPK values were taken at the time of admission, repeated after 48 and 96 hours, and correlated with clinical outcomes and the POP scale. CPK was measured using the VITROS CPK microslide method, and pseudocholinesterase/butyrylcholinesterase (BChE) was measured using the VITROS cholinesterase microslide method. The data were presented as median interquartile range (IQR). Friedman’s two-way analysis of variance by ranks served to compare repeated measures within groups over time, the Mann-Whitney U test served to compare differences between two independent groups, the Kruskal-Wallis test served to compare medians across groups, and the Spearman correlation coefficient served to evaluate relationships among the variables.

Observations and results: The POP scores revealed that serum CPK levels rose as the degree of poisoning increased. The median (IQR) CPK level at admission in the mild group was 240 (180-360) IU/L, the median in the moderate group was 433 (366-507) IU/L, and the median in the severe group was 1,122 (939-1,235) IU/L. Conversely, as the severity of the poisoning increased, the total atropine required increased, but serum levels of BChE decreased. Serum CPK levels and the POP scale showed a strong positive correlation (Spearman’s rho=0.831, p=0.0001). Serum CPK levels and the total atropine requirement were also found to be moderately positively correlated (Spearman’s rho=0.311, p=0.016). On the other hand, serum CPK levels showed a strong negative correlation with pseudocholinesterase levels (Spearman’s rho=-0.631, p=0.00000007). There was also a strong positive correlation between serum CPK at admission and the duration of hospital stays (Spearman’s rho=0.84, p<0.0001). The CPK in the patients who required ventilatory support, 419.5 (243.8-697.2), was higher than in patients who did not require ventilatory support, 386.5 (243.8-545.0), p=0.98. The median (IQR) of the initial CPK levels in the patients who died, 1,288 (1,252-1,292), was significantly higher than in the patients who survived, 378 (238-521) (p=0.0001).

Conclusion: Increased severity of OP poisoning as measured by the POP score, the requirement for higher doses of atropine, reduced levels of BChE, longer hospital stays, a greater need for ventilatory support, and a higher risk of death were associated with elevated serum CPK levels at the time of admission. Therefore, the estimation of serum CPK on admission is advisable in patients with OP poisoning because serum CPK can reliably indicate severity.

## Introduction

India is a developing country, and most of its population is directly or indirectly dependent on agriculture. Organophosphorus (OP) pesticides are widely used for crop protection and pest control, and their ubiquity, availability, and low cost make accidental exposure common among farmers; these pesticides are also frequently used for self-poisoning [1]. In fact, deliberate self-harm through the ingestion of these toxic compounds is a major public health concern, especially in developing countries [2]. Thus, according to the National Poison Information Centre in New Delhi, OP is one of the most frequently encountered toxic agents in cases of suicidal poisoning [3]. Gunnell et al. estimated that there were 258,234 deaths annually from OP poisoning worldwide, accounting for about 30% of suicidal cases [4]. According to Amin et al., organophosphate compounds (OPCs) are responsible for more than 75% of all acute poisoning cases in hospital practice in Bangladesh, where the death rate is approximately 16% [5].

OPCs act by phosphorylating the serine hydroxyl residue on the acetylcholinesterase enzyme, thereby leading to the accumulation of acetylcholine. This accumulation results in cholinergic symptoms, which can be classified as either peripheral or central. The overall toxicity depends on the dose received, the route of administration, the type of OPC, and other individual factors, with OPC poisoning ranging from short- to long-term and from mild to severe [6]. Peripheral symptoms include vomiting, miosis, diarrhea, muscle fasciculations, urinary incontinence, bronchoconstriction, and central effects, including respiratory depression and delirium [7]. The most common cause of death in OP poisoning is respiratory failure, which may result from central or peripheral mechanisms. Studies indicate that the primary mechanisms leading to respiratory failure in OP poisoning are of central origin [8]. The vagus nerve plays a key role in connecting the brain and lungs by providing mechanoreceptor feedback. This vagal mechanism influences hypoventilation and pulmonary secretions, which are commonly observed in OP poisoning cases [8]. OP poisoning also increases the respiratory workload through changes in pulmonary compliance and the obstruction of airways [9]. Creatine phosphokinase (CPK) is an enzyme found in many tissues that converts phosphocreatine to creatine, so it is a biomarker for muscle injury. Fatalities from OP poisoning often result from delays in diagnosis or improper management. The key to survival is early diagnosis, rapid decontamination, and definitive therapy, which fall under the expertise of emergency medicine.

There is no universally accepted scoring system for assessing the severity or predicting the outcome of OP poisoning. The prognosis depends on several factors, including the dose and toxicity of the OP ingested (e.g., neurotoxicity potential, half-life, and rate of aging), conversion into toxic metabolites (e.g., of parathion to paraoxon), and chemical structures (dimethyl or diethyl OP compounds). The Peradeniya Organophosphorus Poisoning (POP) scale is a simple and effective system for determining the severity of OP poisoning. This scoring system was introduced by Senanayake and Keralliedde in 1993 [10]. Common clinical manifestations of OP poisoning serve as parameters (i.e., pupil size, respiratory rate, heart rate, fasciculations, level of consciousness, and seizures), each being assessed on a three-point scale ranging from 0 to two. The score, which represents the muscarinic, nicotinic, and central effects of the acute cholinergic manifestations of OP poisoning, is obtained at initial presentation before any medical intervention. An overall score of 0 to three is considered mild poisoning, a score of four to seven is considered moderate poisoning, and a score of eight to 11 is considered severe poisoning [10].

Laboratory evidence of OP poisoning is usually confirmed by measuring decreases in pseudocholinesterase/butyrylcholine esterase (BChE) and acetylcholine esterase (AChE) activities. However, these tests are costly and not performed regularly in low-resource setting laboratories. There are emerging options for cheaper and/or easily quantifiable biochemical indicators of OP poisoning, such as CPK, lactate dehydrogenase (LDH), and serum immunoglobulins (IgG, IgA), but immunoglobulin assays are costly and difficult to perform. In several animal model studies, the serum level of CPK is often elevated in OP poisoning, so CPK may be used as a biomarker [11].

The aim of this study was to evaluate serum CPK as a predictor of the severity of OP poisoning and its correlation with the POP scale through clinical assessment and categorization of OP poisoning cases on admission based on the scale. The serum CPK levels in OP poisoning cases were estimated at admission and serially at 48 and 96 hours and analyzed for correlations with the POP scale, pseudocholinesterase levels, the requirement for atropine, and the outcomes of the disease.

## Materials and methods

Study design

This was a hospital-based observational study conducted at the Department of General Medicine at the Kalinga Institute of Medical Sciences, Bhubaneswar, India, over 18 months, from September 2018 to August 2020.

Ethical considerations

The study protocol was approved by the Institutional Ethics Committee of Kalinga Institute of Medical Sciences (approval number: KIMS/KIIT/IEC/145/2018), and informed consent was obtained from all of the conscious patients and from the relatives of the unconscious patients.

Study criteria

All cases of OP poisoning reaching the hospital within 12 hours of consumption were included in the study. Patients with chronic liver disease, chronic kidney disease, myopathy, malignancy, myocardial infarction, or myocarditis; those who were taking drugs such as statins, furosemide, aspirin, or intramuscular injections; and those who did not give consent were excluded.

Procedure

After initial resuscitation of the patients, the samples were collected aseptically to evaluate the levels of serum CPK and serum cholinesterase. The severity of poisoning was initially assessed using the POP scale (Table 1). The assessments were graded as mild POP scores (0 to three), moderate POP scores (four to seven), or severe POP scores (eight to 11) (Table 1) [10].

**Table 1:** Peradeniya Organophosphorus Poisoning (POP) scale 0 to three: mild poisoning; four to seven: moderate poisoning; eight to 11: severe poisoning; bpm: beats per minute Source: [10]

Parameters	Criteria	Score
Pupil size	≥2 mm	0
	<2 mm	1
	Pin point	2
Respiratory rate	<20 cpm	0
	≥20 cpm	1
	≥20 cpm with central cyanosis	2
Heart rate	>60 bpm	0
	41-60 bpm	1
	<40 bpm	2
Fasciculations	None present	0
	generalised/continuous	1
	Both generalised and continuous	2
Level of consciousness	Conscious and rational	0
	Impaired response to verbal command	1
	No response to verbal command	2
Seizures	Absent	0
	Present	1

Serum CPK levels were repeated at 48 and 96 hours after admission along with clinical evaluation of the patients. Intramuscular injections were avoided during the course of treatment.

Tools and assessments

CPK was measured using the VITROS CPK microslide method, which measures the conversion of creatine phosphate to ATP and creatine. This assay is sensitive from 20 to 1,600 IU/L of total CPK activity. For samples that were out of range, sample dilution was performed. This assay is precise, with a 2.4% coefficient of variation compared with other VITROS systems. BChE was measured using the VITROS cholinesterase microslide method, for which BChE serves as a substrate. To guarantee accuracy and dependability, both internal and external quality controls were used throughout the essay process. The internal controls used samples with known values to run alongside patient specimens, and the external evaluations involved proficiency testing programs to verify performance against established standards.

Sample size

All serial cases of OP poisoning in the Department of General Medicine at Kalinga Institute of Medical Sciences during the study period were included in the sample.

Statistical analysis

The quantitative data were presented as median (interquartile range (IQR)), and the qualitative data were presented as frequency and percentage tables. A normality check of continuous variables was done using the Shapiro-Wilk test. The Kruskal-Wallis H test was used to compare the median across multiple groups, Friedman’s two-way analysis of variance by ranks was used to compare repeated measures within groups over time, the Mann-Whitney U test was used to compare differences between two independent groups, and Spearman’s correlation coefficient was used to evaluate relationships between variables. P-values of less than 0.05 were considered statistically significant. Microsoft Excel 2017 (Microsoft Corp., Redmond, WA) and IBM SPSS Statistics for Windows, version 20.0 (IBM Corp., Armonk, NY) were used for the statistical analysis.

## Results

Among the patients, 38.3% were 21-30 years of age, 26.7% were 31-40, 15% were 41-50, 10% were <20, 6.7% were 51-60, and 3.3% were >60 years. The median (IQR) age of the patients was 31 years (14-62 years) (16.8) years. Forty-five of them (75%) were male, and 15 (25%) were female; thus, there was male preponderance, and the male:female ratio was 3:1. Nineteen of the patients (31.7%) resided in urban areas and 41 (68.3%) in rural areas. Eighteen (30%) were laborers, 13 (21.7%) were farmers, and 11 (18.3%) were housewives. Nearly half (48.3%) were from the lower class, followed by those from the lower middle class (26.7%), upper middle class (15%), and upper class (10%). Twenty-five (41.6%) had a primary education, 19 (31.7%) had a high school education, four (6.7%) had post-graduate training, and 12 (20%) were illiterate. Most of the patients (96.7%) had consumed the poison intentionally, while only two (3.3%) had been exposed accidentally. The most common symptoms were nausea/vomiting (85%), followed by abdominal cramps (46.7%), excessive salivation (36.7%), cough (31.7%), urinary incontinence (28.3%), diarrhea (23.35%), chest tightness (13.3%), and convulsions (3.3%). Most of the patients presented with miosis (91.75%); other symptoms included bradycardia (38.3%), tachypnea (35%), altered sensorium (30%), fasciculations (10%), hypertension (8.3%), tachycardia (5%), and hypothermia (3.3%). The most common OP compound consumed was chlorpyriphos (33.4%), followed by methyl parathion (28.3%), monocrotophos (20%), triazophos (8.35%), malathion (5%), dimethoate (3.3%), and dichlorvos (1.7%), as Table 2 shows.

**Table 2:** Demographic data of the participants

Parameters	Variables	n (%)
Age (years)	<20	6(10%)
	21-30	23(38.3%)
	31-40	16(26.7%)
	41-50	9(15%)
	51-60	4(6.7%)
	>60	2(3.3%)
Sex	Male	45(75%)
	Female	15(25%)
Urban/Rural	Urban	19(31.7%)
	Rural	41(68.3%)
Occupation	Labourer	18(30%)
	Farmer	13(21.7%)
	Housewife	11(18.3%)
	Service	10(16.7%)
	Student	8(13.3%)
Socio-economic status	Lower	29(48.3%)
	Lower middle	16(26.7%)
	Upper middle	9(15%)
	Upper	6(10%)
Education	Upto primary level	25(41.6%)
	High school	19(31.7%)
	Graduate	4(6.7%)
	Illiterate	12(20%)
Reason	Intentional	58(96.7%)
	Accidental	2(3.3%)
Agents	Chlorpyriphos	20(33.4%)
	Methyl parathion	17(28.4%)
	Monocrotphos	12(20%)
	Triazophos	5(8.3%)
	Malathion	3(5%)
	Dimethoate	2(3.3%)
	Dichlorvos	1(1.7%)

Most of the patients (51.7%) were admitted to the hospital <6 hours after ingesting OP poison, while 22 (36.6%) were admitted within six to 12 hours after ingestion, and seven (11.7%) were admitted >12 hours after ingestion.

On the POP scale, 26 patients (43.3%) had mild poisoning, while 21 (35%) had moderate poisoning and 13 (21.7%) had severe poisoning.

The median (IQR) of the pseudocholinesterase and serum CPK values were 501 (340-1936) IU/L and 401 (240-557) IU/L, respectively. The median (IQR) of atropine required was 399 (290-475) mg, as Table 3 shows.

**Table 3:** Baseline findings of the patients at admission IQR: interquartile range; CPK: creatinine phosphokinase

Parameters	Median	IQR	Min-Max
Pseudo cholinesterase(IU/L)	501	340-1936	60-8172
Serum CPK(IU/L)	401	240-557	93-1292
Atropine(mg)	399	290-475	219-600

The levels of pseudocholinesterase at admission were 1,406 (609-5,701) IU/L in the mild group, 448 (382-2,689) IU/L in the moderate group, and 260 (210-341) IU/L in the severe group. There was a significant increase in the median (IQR) of the pseudocholinesterase value with treatment in the mild group, from 1,406 (609-5,701) IU/L at admission to 2,324 (2,192-2,386) IU/L at 48 hours and 2,070 (2,000-2,294) IU/L at 96 hours. Similarly, there was a significant increase in the median (IQR) of the BChE value in the moderate group, from 448 (382-2,689) IU/L at admission to 882 (779-1,032) IU/L at 48 hours and 800 (730-850) IU/L at 96 hours. The BChE value in the severe group was 260 (210-341) IU/L at admission, 240 (223-252) IU/L at 48 hours, and 255 (225-266) IU/L at 96 hours. The increase in the BChE value with treatment in the mild and moderate groups was significant (p=0.006 and 0.013, respectively), while the changes in the severe group were not statistically significant (p=0.368).

The median (IQR) of the serum CPK values of the patients decreased significantly, from 401 (240-557) IU/L at admission to 308 (214-393) IU/L at 48 hours and 218 (145-412) IU/L at 96 hours. There was a significant decrease in the serum CPK values from admission to 96 hours with treatment. The median (IQR) values of the atropine required in the mild, moderate, and severe groups were 333 (284-430) mg, 428 (272-530) mg, and 450 (398-547) mg, respectively. The median (IQR) of the initial CPK values in the mild, moderate, and severe groups were 240 (180-360) IU/L, 433 (366-507) IU/L, and 1,122 (939-1,235) IU/L, respectively, while the median (IQR) pseudocholinesterase values in the mild, moderate, and severe groups were 1,406 (609-5,701) IU/L, 448 (382-2,689) IU/L, and 260 (210-341) IU/L, respectively. There was a significant association between the POP scale, the initial CPK and pseudocholinesterase levels, and the atropine required, according to the results of the Kruskal-Wallis H test (p<0.05), as Table 4 shows.

**Table 4:** Comparison of the initial serum creatine phosphokinase (CKP) and pseudocholinesterase levels and atropine requirement across the Peradeniya Organophosphorus Poisoning (POP) scale

At admission		POP scale		Kruskal-Wallis H test	p- value
	Mild	Moderate	Severe		
Serum CPK (IU/L)	240(180-360)	433(366-507)	1122(939-1235)	41.568	<0.001
Pseudocholinesterase	1406(609-5701)	448(382-2689)	260(210-341)	25.709	<0.001
Atropine	333(284-430)	428(272-530)	450(398-547)	9.339	0.009

**Figure 1:**
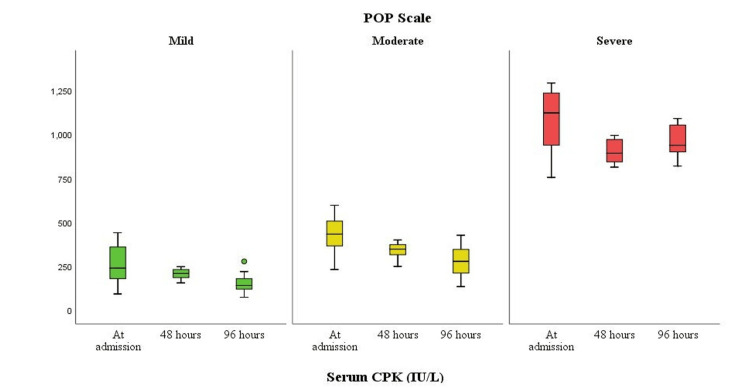
Box plot comparison of serum creatine phosphokinase (CPK) levels over time in the Peradeniya Organophosphorus Poisoning (POP) scale severity groups

There was a significant reduction in the CPK values in the mild group, from 240 (180-360) IU/L at admission to 210 (186-232) IU/L at 48 hours and 142 (120-180) IU/L at 96 hours. There was also a significant reduction in the CPK values in the moderate group, from 433 (366-507) IU/L at admission to 348 (316-373) IU/L at 48 hours and 278 (212-346) IU/L at 96 hours. Likewise, the reduction in the CPK values in the severe group was significant, from 1,122 (939-1,235) IU/L at admission to 893 (844-971) IU/L at 48 hours, but the values increased to 938 (901-1053) IU/L at 96 hours. The overall reduction in the CPK values with treatment in the mild and moderate groups was significant (p=0.0002 and p=0.0004, respectively), while the overall change in the severe group was not statistically significant (p=0.1462), as Table 5 and Figure 1 show.

**Table 5:** Comparison of serum creatine phosphokinase (CPK) levels over time in the Peradeniya Organophosphorus Poisoning (POP) scale severity groups

POP scale	Serum CPK (IU/L)			Test statistic	p-value
	At admission	48 hours	96 hours		
Mild	240 (180-360)	210 (186-232)	142 (120-180)	17.154	0.0002
Moderate	433 (366-507)	348 (316-373)	278 (212-346)	15.687	0.0004
Severe	1122 (939-1235)	893 (844-971)	938 (901-1053)	3.846	0.1462
Related-samples Friedman’s two-way analysis of variance by ranks					

The duration of hospital stays was <7 days for nine patients (15%), seven to 14 days for 33 patients (55%), and >14 days for 18 patients (30%). There was a positive correlation between initial serum CPK and the length of hospital stays (Spearman’s rho=0.840, p<0.0001). Twenty-two patients (36.7%) required ventilatory support. The median (IQR) of the CPK values in these patients was 419.5 (243.8-697.2), higher than in those who did not require ventilatory support, which was 386.5 (243.8-545.0; p=0.98). Fifty-seven patients (95%) survived, while three (5%) died during our study. The median (IQR) of the initial CPK levels in the patients who survived, 1,288 (1252-1292) IU/L, was significantly higher than that in those who died, 378 (238-521) IU/L, p=0.0001.

**Figure 2:**
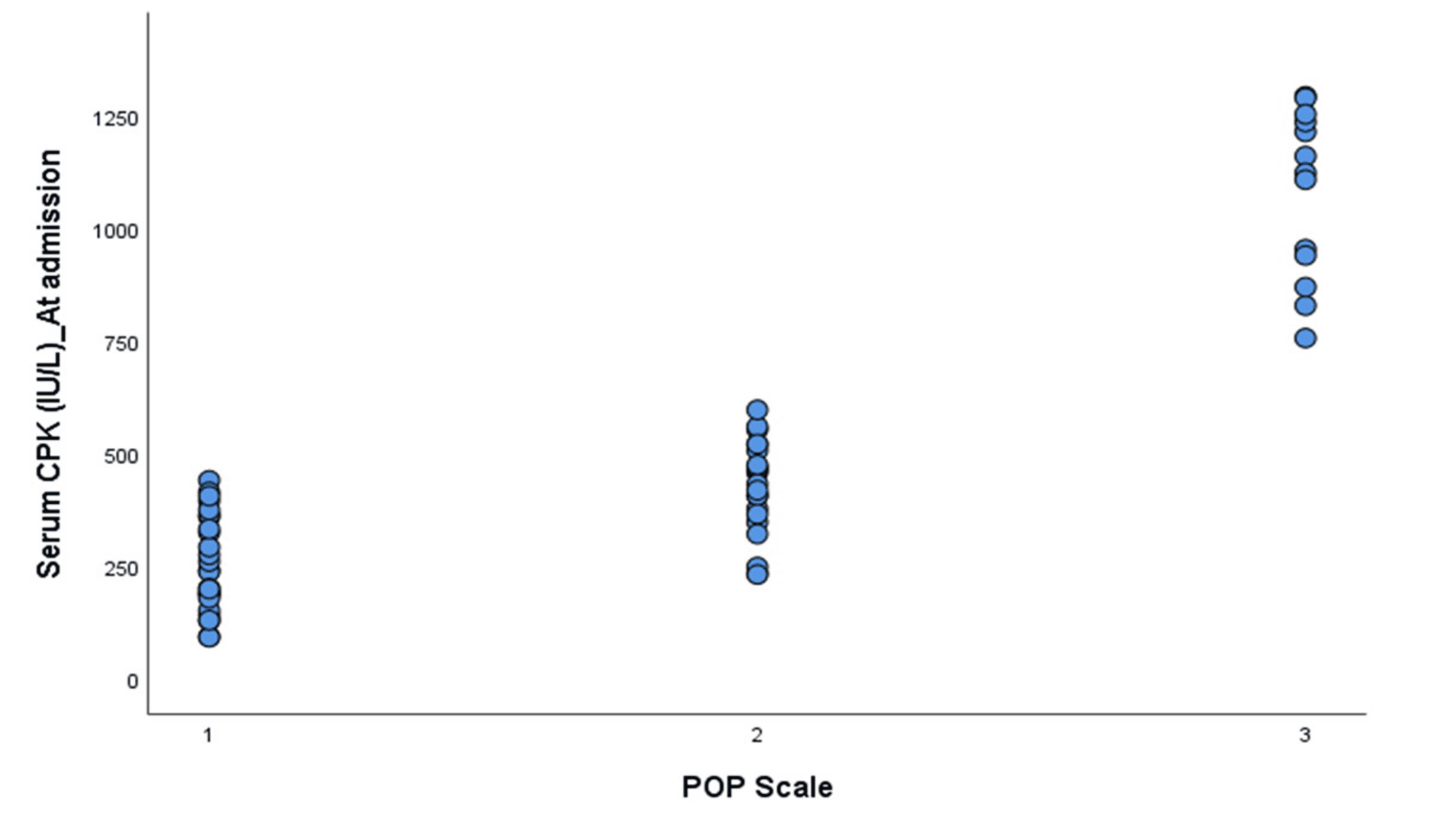
Jitter plot showing the association between serum creatine phosphokinase (CKP) at admission and the Peradeniya Organophosphorus Poisoning (POP) scale 1: mild POP scale (0 to three); 2: moderate POP scale (four to seven); 3: severe POP scale (eight to 11)

**Figure 3:**
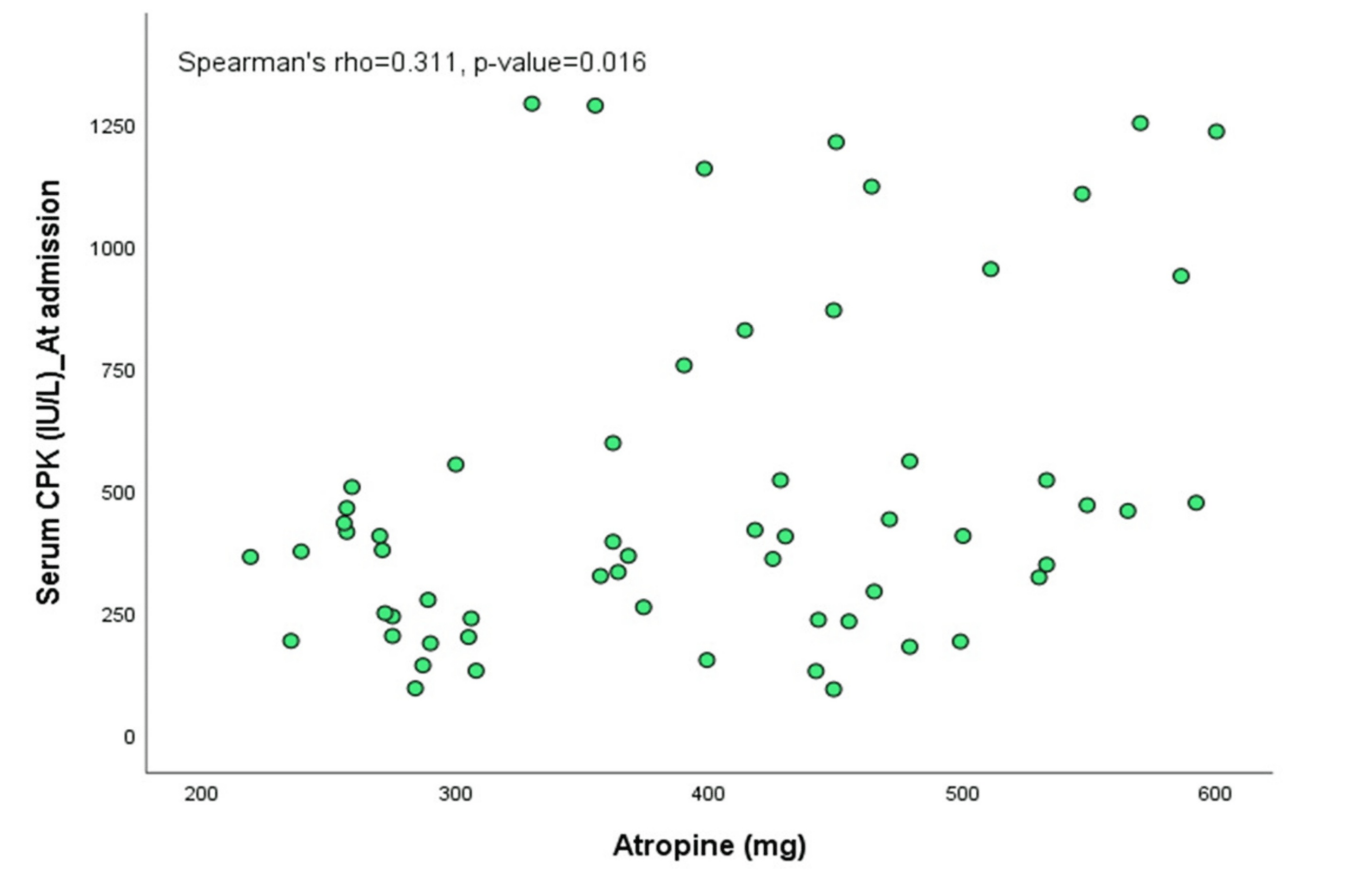
Scatter plot showing association between serum creatine phosphokinase (CKP) at admission and atropine requirement

**Figure 4:**
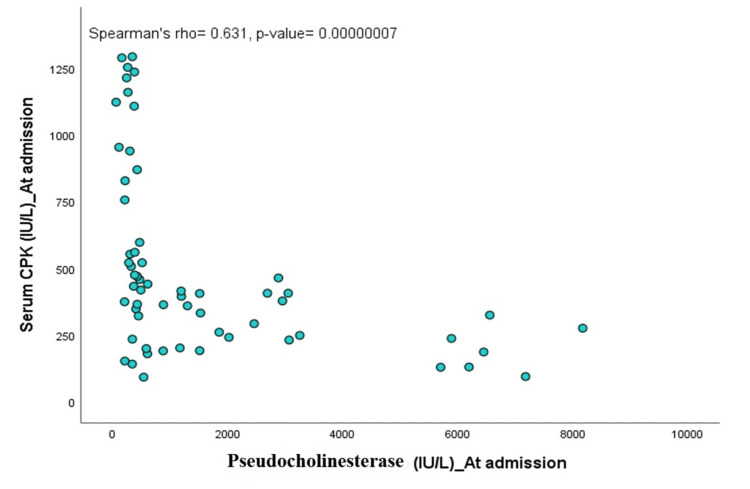
Scatter plot showing the association of serum creatine phosphokinase (CKP) and pseudocholinesterase level at admission

There were significant positive correlations between serum CPK levels and the POP scale (Spearman’s rho=0.831, p=0.0001) and between serum CPK values and the dose of atropine required (Spearman’s rho=0.311, p=0.016). A significant negative correlation was observed between serum CPK levels and pseudocholinesterase levels (Spearman’s rho=-0.631, p=0.00000007), as shown in Figures 2-4.

## Discussion

This hospital-based observational study was conducted with 60 patients to analyze the serum CPK level as a prognostic marker for OP poisoning and its correlation with the POP scale. OP compounds are commonly used pesticides in agriculture, and because of their easy accessibility and wide use, poisoning with these compounds has emerged as an important health concern, especially in developing countries.

Acute OP poisoning can manifest in three distinct phases of toxicity: acute cholinergic crisis, intermediate syndrome (IMS), and OP-induced neuropathy (OPIDN). The muscarinic features include excessive salivation, lacrimation, and urination, as well as diarrhea, gastrointestinal cramps, emesis, blurred vision, miosis, bradycardia, and wheezing. The nicotinic features include fasciculation, paresis, and paralysis. The central receptor features include anxiety, confusion, psychosis, seizures, and ataxia [12]. IMS is termed “intermediate” because it occurs in the interval between the end of the acute cholinergic crisis and OPIDN. Studies have shown that IMS occurs 48 and 96 hours after acute poisoning, is characterized by weakness in the proximal limb muscles, neck flexors, and respiratory muscles, and is attributable to muscle fiber necrosis. Reversible myocyte injury increases muscle enzymes such as myoglobin, LDH, troponin, and CPK. Serum CPK rises within six hours following muscle injury and remains elevated for five to six days [13].

Among the patients in the present study, 38.3% were 21-30 years of age, 26.7% were 31-40, 15% were 41-50, 10% were <20 years, 6.7% were 51-60, and 3.3% were >60. The median (IQR) age of the patients was 31 (16.8) years. Forty-five of them (75%) were male, and 15 (25%) were female. There was a male preponderance, and the male:female ratio was 3:1. This demographic profile is similar to those in previous studies [14-17]. Further, 19 patients (31.7%) resided in urban areas, and 41 (68.3%) lived in rural areas; 18 of them (30%) were laborers, 13 (21.7%) were farmers, and 11 (18.3%) were housewives; and 10 (16.7%) were in service, and eight (13.3%) were students. This demographic profile is comparable to those in previous studies [14-15]. According to the POP scale, 26 of the patients in the present study (43.3%) had mild poisoning, 21 (35%) had moderate poisoning, and 13 (21.7%) had severe poisoning. This finding is also consistent with the findings of previous studies [16-18].

The serum CPK values decreased significantly from admission to 96 hours with treatment in the present study. More specifically, CPK values decreased in the mild and moderate groups from admission to 48 hours and 96 hours, while the decrease in the severe group was significant from admission to 48 hours, but there was no decrease in the CPK values in this group at 96 hours. The decrease in CPK values with treatment was significant in the mild and moderate groups, while the changes in the severe group were not statistically significant. This finding is consistent with that reported in a previous study [16].

The levels of pseudocholinesterase decreased as the severity of OP poisoning increased. The increase in pseudocholinesterase values with treatment in the mild and moderate groups was significant, while the changes in the severe group were not statistically significant. These findings are consistent with those of previous studies [14, 17]. In a prospective, observational study, the statistical comparison relation was found to be highly significant (p<0.0001), showing that low serum cholinesterase value was associated with higher mortality [16]. In the present study, the follow-up serum CPK levels measured in recovering patients without any complications during the therapy course tended to decrease, but the patients who were severely poisoned developed complications during the therapy, especially seizures, and acidosis remained elevated. A previous study reported that, of 59 patients, 38 had increasing BChE levels, and 21 had decreasing BChE levels [17]. In the present study, none of the patients with increasing BChE died, but five with decreasing BChE expired, and this result was statistically significant (P=0.0080).

In the present study, the initial CPK levels were significantly higher in the patients who died than in those who survived. High CPK values were also associated with ventilatory requirements. The higher median in the ventilated group indicates a trend toward severe neuromuscular involvement, though the interquartile ranges overlapped. This finding lends credence to the idea that CPK may serve as an early indicator of respiratory failure in cases of OP poisoning. Despite the lack of statistical significance, the clinical implications are significant, particularly when it comes to directing intensive care unit admissions and predicting ventilatory requirements. Proactive respiratory support may result in early detection in these patients, which could lower morbidity and enhance the results of treatment. Higher CPK levels were also linked to noticeably longer hospital stays.

There was a significant positive correlation between the serum CPK levels and the POP scale in the present study, as well as between the serum CPK values and the dose of atropine required, while a significant negative correlation was observed between the serum CPK levels and the pseudocholinesterase levels. Further, as the severity of the poisoning increased with respect to the POP scores, the serum CPK levels and the total dose of atropine required for treatment also increased, but the levels of BChE decreased. Similar observations were made in previous studies [14, 16, 18].

There was a significant association between the POP scale values, the atropine requirement, initial CPK levels, and pseudocholinesterase levels (p<0.05). Similarly, a previous study of the role of CPK as an alternative prognostic marker and the relationship between CPK levels and the POP scale values for OP poisoning reported that serum CPK, BChE levels, and the total atropine dose strongly correlated with clinical severity [18].

In cases of ongoing injury to muscle resulting from the development of complications, CPK levels remain elevated since the half-life of CPK is about 1.5 days, and it normalizes within five to six days of a single insult to the muscle [13]. When skeletal muscle is injured, CPK leaks into the blood and urine. Accordingly, the serum CPK level remains the best biomarker for detecting and monitoring skeletal muscle damage and disease [19]. The main disadvantage of serum CPK as a biomarker is its non-specificity, which necessitates the exclusion of other conditions.

There is scope for large-scale, multicentric studies to assess the role of serum CPK as an indicator of severe OP compound exposure.

## Conclusions

The severity of OP poisoning, as measured by the POP score, an increased requirement for atropine, low BChE levels, the length of hospital stays, the need for ventilatory support, and the risk of death, was associated with elevated serum CPK levels among the patients in this study at the time of admission. More specifically, the results of the study suggest that there is a significant correlation between serum CPK and POP scale values, the requirement for atropine, and pseudocholinesterase levels. These results validate the use of initial serum CPK as a biomarker for prognosis and, potentially, as a triage tool. Serum CPK can serve as a relatively inexpensive and easily quantifiable indicator of OPC exposure. Accordingly, consideration should be given to routinely estimating serum CPK at admission for use as a reliable measure of the severity of OP poisoning and a prognostic marker.
